# Demonstration of Redox Potential of *Metschnikowia koreensis* for Stereoinversion of Secondary Alcohols/1,2-Diols

**DOI:** 10.1155/2014/410530

**Published:** 2014-01-27

**Authors:** Vachan Singh Meena, Linga Banoth, U. C. Banerjee

**Affiliations:** Department of Pharmaceutical Technology (Biotechnology), National Institute of Pharmaceutical Education and Research (NIPER), Sector 67, S. A. S. Nagar, Punjab 160 062, India

## Abstract

The present work reports the *Metschnikowia koreensis*-catalyzed one-pot deracemization of secondary alcohols/1,2-diols and their derivatives with in vivo cofactor regeneration. Reaction is stereoselective and proceeds with sequential oxidation of (*R*)-secondary alcohols to the corresponding ketones and the reduction of the ketones to (*S*)-secondary alcohols. Method is applicable to a repertoire of racemic aryl secondary alcohols and 1,2-diols establishing a wide range of substrate specificity of *M. koreensis*. This ecofriendly method afforded the product in high yield (88%) and excellent optical purity (>98% *ee*), minimizing the requirement of multistep reaction and expensive cofactor.

## 1. Introduction

Enantiomerically pure secondary alcohols are used as pharmaceuticals, flavors, agricultural chemicals, synthetic intermediates, chiral auxiliaries, and analytical reagents [1]. These enantiopure alcohols can be obtained by kinetic resolution, asymmetric reduction of ketones, oxidation of olefines, ring opening of glycidol with phenol, or stereoinversion of racemic alcohols [2–20]. Among these various methods, stereoinversion is the most promising technique which offers a 100% conversion from racemate to the enantiopure product [21–24].

Sequential chemical oxidation reduction with one or two biocatalysts has been reported in the literature for stereoinversion of racemic alcohols [21, 25–32]. However, the chemical process needs harsh reaction conditions. Combination approach of transition metal catalyst and biocatalyst for stereoinversion of secondary alcohols was also used by various workers [33]. The whole cell biocatalytic stereoinversion is an efficient method for obtaining chiral secondary alcohols [34–37]. Hummel and Riebel detailed the stepwise route to synthesize enantiomerically pure alcohols from the corresponding racemates by employing two stereo complementary alcohol dehydrogenases [38]. The stereoinversion of sec-alcohols by oxidoreductases has also been reported [23]. However, the external addition of the cofactors and the use of isolated or commercially purified enzymes, specific substrates, and moderate substrate concentrations are the limitations of these protocols [36]. Comparing with the above-mentioned approaches, the application of the whole cell biocatalysts for stereoinversion seems to be the more favorable approach in the context of reaction conditions, enzyme stability, and cofactor regeneration. There are only limited reports on the stereoinversion of secondary alcohols using whole cell biocatalysts, such as sec-alcohols and 1,2-diols [30, 36, 39–41]. Obtaining an enantiomerically pure isomer in a one-pot process is currently a hot topic and of great industrial demand [42, 43].

## 2. Material and Methods 

### 2.1. Chemicals

(±)-Phenyl glycidyl ether, (±)-1-phenyl ethanol, (*R*)/(*S*) 1-phenyl ethanol, and acetophenone were purchased from Sigma (Steinheim, Germany). (*RS*) (±)-3-phenoxy-1,2-propanediol and (*S*)-3-phenoxy-1,2-propanediol were synthesized chemically from phenyl glycidyl ether by the reported procedure [44]. Solvents required for the synthesis and extraction were acquired from commercial sources and they were either of analytical or commercial grades obtained from Rankem (Mumbai, India) and Merck Ltd (Whitehouse Station, NJ, USA). Growth media components were obtained from Hi-Media Inc. (Mumbai, India). Various HPLC grade solvents *n*-hexane, 2-propanol and acetonitrile were obtained from J. T. Baker (Phillipsburg, NJ, USA). Membrane filters of 0.22 *μ*M were purchased from MDI Pvt. Ltd. (Ambala, India). All other chemicals used were of analytical grade and obtained from standard companies.

### 2.2. Microorganism and Cultivation Conditions

*Metschnikowia koreensis* MTCC-5520 was used in this study. The strain was isolated in our laboratory, identified by Microbial Type Culture Collection and Gene Bank, Institute of Microbial Technology, Chandigarh, India. 

*Culture on Agar Plate*. The stock culture was maintained at 4°C on agar plate containing YPD medium. The composition of YPD medium was yeast extract (5 g/L), peptone (5 g/L), and dextrose (10 g/L).

*Preculture*. A single colony from the agar plate was aseptically inoculated into 25 mL YPD medium and grown at 25°C (200 rpm) for 24 h.

*Growth of Cellmass*. Five milliliters of preculture was transferred in 100 mL YPD medium in a 500 mL shake flask and incubated at 25°C (200 rpm) for 2 days. The cells were harvested by centrifugation at 10,000 g for 10 min and thoroughly washed. The cells were suspended in Tri-HCl buffer (pH 8) and directly used for biotransformation reaction.

### 2.3. Biotransformation Conditions

#### 2.3.1. Concurrent Oxidation-Reduction of Secondary Alcohols

Typical procedure for deracemization of racemic secondary alcohols to single enantiomer (*S*) with tandem biocatalysts was optimized. one gram wet cellmass of *M. koreensis* was suspensioned in 5 mL Tris-HCl buffer (50 mM; pH 8.0). Racemic secondary alcohols were added into the cell mass suspension to make the final concentration 5 mM in the reaction mixture and reaction was carried out for up to 3 days. The reaction mixture was incubated for fixed time at 30°C (200 rpm). The cells were removed by centrifugation at 10,000 ×g for 10 min and aqueous phase was subjected to reversed phase chiral HPLC analysis for quantifying the reactant and product concentrations.

#### 2.3.2. Reaction Temperature

In order to optimize temperature [45], reaction was performed at different temperatures ranging from 20 to 40°C. Cellmass suspension (150 mg/mL) was prepared in Tris-HCl buffer pH 8, 50 mM and added to the reaction mixture. The stereoinversion was carried out with 5 mM (±)-3-phenoxy-1,2-propanediol as substrate and incubated at various temperatures (200 rpm). The final reaction volume was 15 mL. The reaction was continued for up to 3 days and aliquot (1 mL) was withdrawn at a regular time interval (24 h) and checked for the conversion and enantiomeric excess in chiral-HPLC.

#### 2.3.3. Cellmass Concentration

In order to study the effect of cellmass concentration [45] on stereoinversion, various cellmass concentrations ranging from 100 to 300 mg/mL were used. All other parameters are kept at their optimal values and checked for stereoinversion by performing the reaction in Tris-HCl buffer (pH 8, 50 mM) with 5 mM (±)-3-phenoxy-1,2-propanediol. The final reaction volume was 15 mL. The reaction was continued for up to 3 days and aliquot (1 mL) was withdrawn at a regular time interval (24 h) and checked for the conversion and enantiomeric excess in chiral-HPLC.

#### 2.3.4. Substrate Concentration

In order to find out the optimum substrate concentration [45], various substrate concentrations ranging from 5 to 20 mM in the reaction mixture were added. Cellmass (250 mg/mL) suspended in Tris-HCl buffer (pH 8, 50 mM) was used to perform this experiment. The reaction was carried out at a final volume of 15 mL at 30°C. The reaction was continued for up to 3 days and aliquot (1 mL) was withdrawn at a regular time interval (24 h) and checked for the conversion and enantiomeric excess in chiral-HPLC.

### 2.4. Analytical Methods

Quantitative formation of single enantiomer of 3-phenoxy-1,2-propanediol, 1-phenyl ethanol, and their corresponding ketones was estimated by High Performance Liquid Chromatography (HPLC, Shimadzu 10AD VP, Kyoto, Japan), equipped with UV detector using a Lux cellulose-1 chiral (4.6 mm × 250 mm, 5 *μ*m, phenomenex, USA) column at 25°C. Elution was carried out by acetonitrile and water (35 : 65) at a flow rate of 0.5 mL/min and detected at 254 nm and 215 nm, respectively.

## 3. Result and Discussion 

**Scheme 1:**
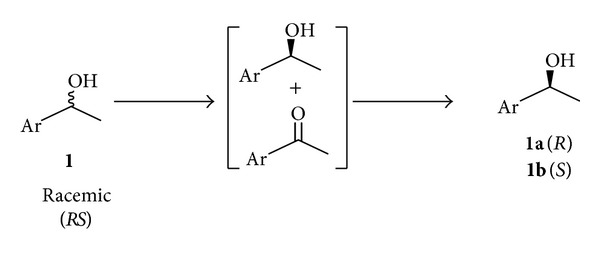
Concurrent oxidation and reduction for the stereoinversion of racemic secondary alcohols.

**Scheme 2:**
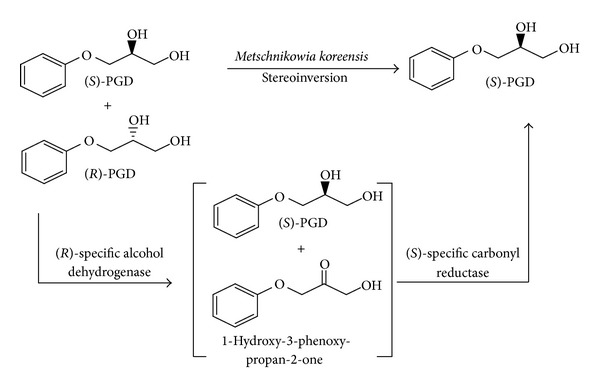
Stereoinversion of 3-aryloxy-1,2-propanediols by the whole cells of *Metschnikowia koreensis. *

In this paper, a single whole cell biocatalyst (one pot) was successfully demonstrated for stereoinversion of aryl secondary alcohols (Scheme 1) and 1,2-diols (Scheme 2) to enantiopure (*S*)-alcohols in excellent yield and enantioselectivity. The present work is an attempt to combine the multienzyme reactions into single-step reactions, while minimizing the conventional drawbacks of catalysis.

*M. koreensis* was examined for its ability to catalyze the stereoinversion process of 1-phenylethanol and 3-aryloxy-1,2-propanediol. The racemic alcohols/diol was converted into single enantiomer, indicating the stereoinversion process catalyzed by redox enzyme. Similar findings were also reported in the literature [41]. It was observed that the whole cells of *M. koreensis* showed good stereoinversion. Sufficiently convinced with the microbial potential of stereoinversion, a detailed systematic optimisation study of various reaction parameters was carried out. The optimum temperature for the stereoinversion process was found to be 30°C. Below and above this temperature, the conversion and enantiomeric excess suffered. The results indicated good enzyme stability and activity at 30°C. Buffers of various pHs ranging from 5 to 8 were tested and it was found that Tris-HCl buffer of pH 8 gave the best results, while keeping the other reaction parameters constant. The optimized cell-mass and substrate concentration were found to be 250 mg/mL and 5 mM, respectively. A mixing rate of 250 rpm was selected as optimum. To study the time course of the *M. koreensis-*catalyzed stereoinversion process, the reaction mixture of racemic 1-phenylethanol was subjected to chiral chromatography at different time intervals. A maximum yield of 98%, with 99% *ee* of (*S*)-after 18 h, was achieved. To investigate mechanistic details of the stereoinversion process, the ketone was used as a model substrate for *M. Koreensis*. The production of (*S*)-alcohol was observed from this reaction [46, 47]. This study suggested a cascade of events that included the initial oxidation of (*R*)-alcohol to ketone in a highly selective reaction leaving (*S*)-alcohol as such. This process is followed by the reduction of ketone to (*S*)-alcohol in higher enantiomeric excess and yield (Table 1).

The biocatalytic stereoinversion behaviour of the 1-phenylethanol derivatives by other microorganisms was also reported in the literature [22, 37]. The encouraging outcome of this study prompted us to test the applicability of this biocatalyst for the stereoinversion of other derivatives of 1-phenylethanol and 3-aryloxy-1,2-propanediol. Impressive results were obtained in each case. Various functional group-substituted alcohols underwent a clean deracemization process and produced (*S*)-isomer with excellent yield and enantiomeric excess (Table 1).

Extrapolation of a similar biocatalytical condition to *M. koreensis-*mediated stereoinversion of (*S*)-3-aryloxy-1,2-propanediol proved the excellent redox potential of this organism towards a diverse array of substrates. It is noted that microbes gave a higher chemical yield with excellent stereoinversion after 3 days of incubation. An overall view of the deracemization process of 3-aryloxy-1,2-propanediol is presented in Table 2.

## 4. Conclusion

In conclusion, we have identified and demonstrated the redox potential of *Metschnikowia koreensis *for the stereoinversion process of secondary alcohols/1,2-diols. Further research may be initiated on finding out the detailed mechanistic investigation, isolation of probable enzymes, and substrate diversification on the application of this stereoinversion process.

## Supplementary Material

M. koreensis-mediated biocatalysis for the stereoinversion of (S)-3-aryloxy-1,2-propanediol proved the excellent redox potential of this organism towards a diverse array of substrates. It is noted that microbes gave a higher chemical yield with excellent stereoinversion after 3 days of incubation. An overall view of the deracemization process of 3-aryloxy-1,2-propanediol is presented here. The supplementary information supported the chiral HPLC resolution and accordingly utilized for calculating the enantiomeric yield.

## Figures and Tables

**Table 1:** Results of the deracemization of secondary alcohols^a^.

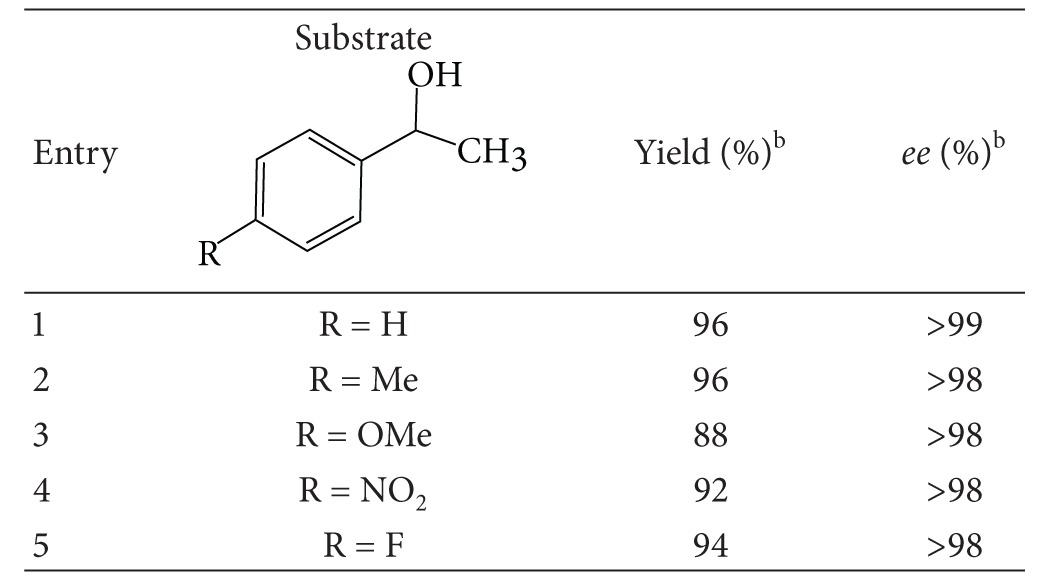

^a^Reactions were carried out with different substrates (5 mM, each) using resting cells (250 mg/mL). ^b^Yield and *ee *were determined by chiral HPLC with Phenomenex Lux cellulose-1 (250 × 4.6 mm) column.

**Table 2:** Biocatalytic deracemization of 3-aryloxy-1,2-propanediol by stereoselective oxidation reduction using whole cells of *M. koreensis* ^a^.

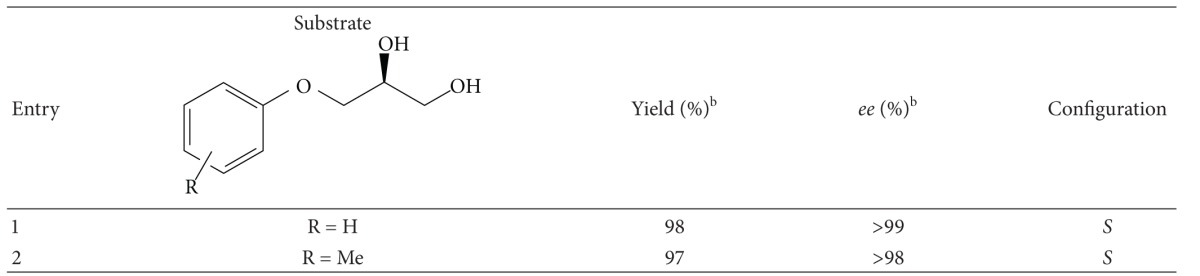

^a^Reactions were carried out with two substrates (5 mM, each) using the resting cells (250 mg/mL). ^b^Yield and* ee* were determined by chiral HPLC with Phenomenex Lux cellulose-1 (250 × 4.6 mm) column.
