# Supplementary material for: Demonstration of Redox Potential of Metschnikowia koreensis for Stereoinversion of Secondary Alcohols/1,2-Diols
Source: Biomed Res Int. 2014 Jan 27;2014:410530. doi: 10.1155/2014/410530 (PMC3921931; doi:10.1155/2014/410530)
Supplement: Supplementary file 1 — M. koreensis-mediated biocatalysis for the stereoinversion of (S)-3-aryloxy-1,2-propanediol proved the excellent redox potential of this organism towards a diverse array of substrates. It is noted that microbes gave a higher chemical yield with excellent stereoinversion after 3 days of incubation. An overall view of the deracemization process of 3-aryloxy-1,2-propanediol is presented here. The supplementary information supported the chiral HPLC resolution and accordingly utilized for calculating the enantiomeric yield. [file 410530.f1.docx]

**Supplementary Information**

Demostration of Redox Potential of *Metschnikowria Korensis* for Deracemization of Secondary Alcohols/1,2-Diols

**Chiral RP-HPLC chromatogram of (±)-3- phenoxy-1, 2-propanediol for deracemization**

**A) 1-days**

**
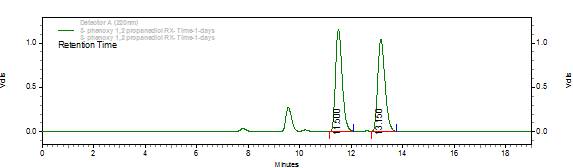
**

R

S

**B) 2-days**


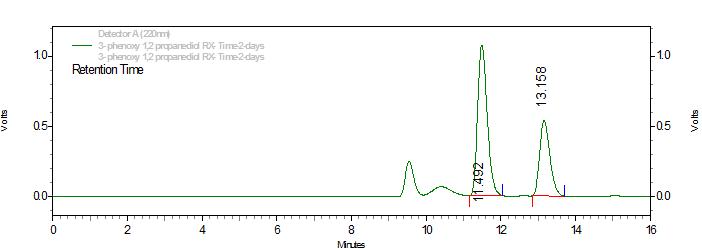


R

S

**C) 3-days**


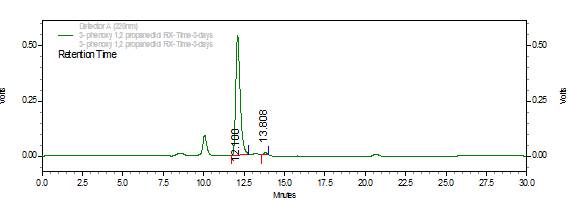


R

S
